# Editorial

**Published:** 1861-01

**Authors:** 


### EDITORIAL.

The American Medical Association.—Under this caption, we find in the *Louisville Monthly Medical Newes*, for November, which has just reached us, an editorial article which closes with the following paragraph:

"The meeting at Chicago, we fear, will be thinly attended. There will doubtless be an excitingly amusing time with the solons of the Garden City, in reference to the next President.

We suppose that even now there is log-rolling and wire-pulling, and feasting, in anticipation of who are to constitute the nominating committee, and who is to be its chairman; for that fact ascertained, who will be President? becomes a foregone conclusion. There are two schools there, and judging from the tone of their respective organs, we would suspect that each had a candidate in the field, for they are playing at cut and thrust, in any other than a christian spirit. We would recommend them to be calm, and beat their swords into ploughshares. There is excitement enough over Illinois Presidents."

We doubt whether another paragraph can be found in print, containing as many false assumptions and unjust intimations, as the above.

1st. It assumes that both of the Medical Journals published in this city are *organs* of the respective Medical Colleges. We take occasion to say that the Chicago Medical Examiner, is as perfectly independant of all medical colleges as any other Journal published in America.

2d. It assumes that the two journals are "playing at cut and thrust, in any other than a *christian spirit.*" We would like to have the editor of the Louisville News, substantiate this by a single line of proof. This assertion would lead his readers to suppose that the Journals and Schools here, were engaged in a bitter and *unchristian* controversy; whereas nothing could be farther from the truth. There is not a line to be found in this Journal for the last six months, which alludes to the Rush Medical College, except in terms of friendship and respect. And on the other side, until its last issue in December 1860, the Chicago Medical Journal, the organ of Rush Medical College, had not so much as informed its readers that the Medical Department of Lind University was in existence.

3d. It assumes that each college has its candidate for the next Presidency of the Association; which is simply an assumption without a shadow of proof.

4ih. It assumes that these candidates and their friends are already "*log-rolling* and *wire-pulling*," to get the next nominating committee properly arranged. Now we assure the editor of the Louisville Medical News, and all other friends of the American Medical Association, that these assumptions concerning the next President of the Association, are not only unqualifiedly false, but unjust to the whole profession of this city and state. It is well known that the senior editor of this Journal, and a large majority of the profession, both of this city and state, are strongly opposed to the custom of confining the selection of President to the place of meeting. So true is this, that the Illinois State Medical Society, at its last annual meeting, passed resolutions, almost unanimously, condemning the practice and instructing its Delegates to use all honorable means to break it up, and to establish the practice of electing all the officers with reference to their fitness without regard to localities. There are two organized Medical Societies in this city, each holding meetings once a month; and although we have attended nearly all the meetings of both, we have never yet heard the subject of the next Presidency of the Association alluded to in either. Our *confrere*

at Louisville, and the members of the Association every-where, may have their minds at perfect ease about "log-rolling" and every other species of strife among the profession in this city. We invited the association to meet here, not for the evanescent honors of its highest office, but for the glorious privilege of receiving its members at our own firesides, of extending to them the same generous hospitality that we have had extended to us in so many other cities, and of permitting them to see, here in the Garden City itself, one of the most striking illustrations of the energy and activity of the age in which we live, that can be found on the continent.

We cordially invite the members and friends of the Association, in every section of the country, in the East, the West, the South, and the North, to honor us with their presence.

We hope that neither political dissensions nor sectional prejudices will deter any from coming up to our annual greeting. We shall look for none with more anxiety; and we are certain that none will receive a more cordial welcome, than those with whom we have so often met from Charleston, Richmond, Baltimore, New-Orleans, Nashville, Louisville, and other cities of the South. Come on friends from all sections; come by hundreds, for we have ample halls, capacious hotels, broad prairies, beautiful lakes, and a generous people. Give us a full meeting, a free interchange of sentiments for the advancement of the noblest and most humane of professions, and the happy privilege of serving you to the best of our ability, and you may confer your Presidential honors on whomsoever you may deem most *worthy*, without the slightest regard to locality, and we will guarantee that the profession of Chicago will be satisfied.

---

To the Members of the Illinois State Medical Society. —We coppy the following characteristic paragraph from the Dec. No. of the *Chicago Medical Journal.* It is evidently from the pen of its Senior editor, Dr. D. Brainard:

"The Transactions of the Illinois State Medical Society, have at last come into being, after a lingering and tedious labor, from among the viscera of the *Examiner*,

We have not yet had time to give the Pamphlet a full reading, but it does strike us as singular, that after such a prolonged gestation, the product shows either "arrest of development" or evisceration. The copies we have seen have omitted eight pages from the body of the report on Surgery. Comment is unnecessary. We shall read the balance at our leisure."

Here we have plainly *insinuated* three distinct charges, so evidently designed to create prejudice against the Permanent Secretary of the State Society, under whose supervision the Transactions are published, that we are not willing to pass them by without a few words of explanation. The three *insinuations* are:

1st. That the publication of the transactions has been usually and improperly delayed.

2d. That such delay was occasioned for the purpose of using the matter first to fill the pages of the Examiner.

3d. That after all the delay, the publication had been so carelessly executed as to leave out "*eight pages* from the body of the report on Surgery."

Instead of characterizing each and all of these charges as wholly gratuitous and false, as we might do with perfect propriety, we will simply submit the following statement of facts. In accordance with the resolutions of the State Society adopted at different times, it has been customary to delay putting the Transactions to press, at least, sixty days after each annual meeting, both for the purpose of giving the Treasurer time to notify non-attending members of the amount of the annual assessment, and of procuring the names of all who pay, for publication. The last annual meeting was held about the middle of May; and on the first of August we had all the papers ready to put to press, except the reports on Surgery by Dr. Brainard, and on Diseases of the Eye by Dr. Holmes. Not anticipating that either of these reports would be of undue length, we sent those in hand to the printer, and notified the gentlemen just named that their papers were wanted for publication. Dr. Holmes promptly sent us his report, but only a part of that on Surgery was given us at first. We had directed the printer to push the work to completion as rapidly as possible ; and to publish no part of it in the Examiner, except the Valedictory Address of Dr. Prince, and the Annual Address. But as the work progressed, and we received the remainder of the report on Surgery, it was ascertained that it alone would make over seventy pages of printed matter ; and would, consequently, swell the volume to a size, much beyond that of any previous year, and also increase the expense beyond the amount in the hands of the Treasurer. The Treasurer was immediately notified of these facts, and with his consent the publication was retarded long enough to enable him to send notices and get returns from such members of the Society as had not yet paid their dues. To lessen the expense as much as possible, we took advantage of this delay, and caused the reports on Obstetrics, Practical Medicine, and Veratrum Viride to be inserted in the Examiner, thereby causing the cost of setting the type for those papers to be charged to that Journal instead of the State Society. The author of the report on Obstetrics also furnished, gratuitously, the wood cuts that accompany that report. Notwithstanding these embarrassments, the Transactions were published, and a copy mailed to each paying member of the State Society early in November ; which is as early as they have been issued in former years. We have twice before relieved the Treasury of the State Society from debt, by publishing a large part of the matter designed for the Transactions in the Medical Journal, and charging the Society only for paper and press-work. If, in endeavoring to prevent the accumulation of another debt this year, by the same means, we have done wrong, let the Society at its next meeting promptly say so ; and we will most cheerfully give them the privilege of choosing a more faithful servant for the future.

In regard to the charge, that the Transactions had been issued in a *mutilated* condition, we have only to say that we have taken the trouble to examine the whole bundle left in our possession, amounting to, at least, 250 copies, without finding one from which a single page was missing. It is *possible* that the *senior editor of the Chicago Medical Journal*

has found a copy, from which the binder had accidentally omitted, in the stitching, a single form of eight pages. If so, his object in publishing the fact to the world is sufficiently obvious. If any member of the State Society, who has paid his dues for the year 1860, has not received his copy of the Transaction, he will confer a favor by notifying me, and he shall be supplied.

N. S. DAVIS,
**Permanent Secretary of Illinois State Medical Society.**

---

Chicago Academy of Medical Sciences.—At the annual meeting of the Chicago Academy of Medical Sciences, held Jan. 4th, 1861. The following fellows were elected officers for the ensuing year:

President, Dr. James Bloodgood. Vice President, Dr. Thomas Bevan. Recording Secretary, Dr. E. L. Holmes. Corresponding Secretary, Dr J. McAlister. Treasurer, Dr. Wm. Scott Denniston. Trustee, Dr. A. Fisher. Committee on Medical Education, Dr. Wm. H. Byford, Dr. J. W. Freer, Dr. Thos. Bevan. Committee on Medical Ethics, Dr. R. C. Hammill, Dr. N. S. Davis, Dr. E. Powell. Committee on Admissions, Dr. J. Wickersham, Dr. H. W. Jones, Dr. S. C. Blake.

---

☞ We would call attention to J. H. Reed & Co's. Catalogue of Surgical Instruments, which we publish in this number of the Journal. Reserve it for future reference.

